# The Influence of the Debunker’s Identity and Emotional Expression on the Sharing Behavior of Debunking Information

**DOI:** 10.3389/fpsyg.2021.783415

**Published:** 2021-12-06

**Authors:** Fan Chao, Xin Wang, Guang Yu

**Affiliations:** School of Management, Harbin Institute of Technology, Harbin, China

**Keywords:** rumor, debunking information, social media, debunker’s identity, textual emotions, crisis management

## Abstract

Owing to the proliferation of rumors on social media, it is necessary to disseminate debunking information to minimize the harm caused by them. Using content analysis, sentiment analysis, and regression analysis, this study examined the mediating role of follower count in the relationship between the debunker’s identity and sharing behavior, and it explored the relationship between the text sentiment of debunking information and sharing behavior based on data on the spread of three rumors that circulated extensively on social media. Using an ordinary account as a reference, we found that the mediating or suppression effect (i.e., when direct and indirect effects are significant and opposite) of follower count in the relationship between debunker’s identity (celebrity, media, or government) and sharing behavior was significant. The three test identities (celebrity, media, and government) had more followers than the ordinary account, which resulted in a significant positive effect on the number of reposts. The debunker’s identity did not have a positive effect on the sharing of debunking information when controlling for mediating variables. Debunking information with emotional overtones (positive or negative) was shared more widely compared with information with neutral emotions, and the dominant emotional polarity was different in the three different rumors. These findings can contribute to the generation of debunking information content, which can aid in the development of effective communication strategies and improvement in the efficiency of crisis management.

## Introduction

The spread of rumors, especially on social media, has had a serious impact on network order and social development (Lazer et al., 2018; Allen et al., 2020). It can cause panic, lead to false accusations, and interfere with the work of emergency response agencies, posing a threat to public safety (Jung et al., 2020). Particularly, since the outbreak of COVID-19, the spread of rumors has become more concerning than the prevention and treatment of the disease itself, causing significant negative consequences (Kassam, 2020). To mitigate the potential harm caused by rumors, it is necessary to dispel them by disseminating debunked information.

Inadequate information to debunk rumors is a key impediment to damage control. For effective debunking, correct information must reach all persons who have encountered the rumors (Wu, 2020). Rumors spread widely to drown out factual as well as retroactive information. One powerful evidence is that messages from the World Health Organization (WHO) and the US Center for Disease Control and Prevention (CDC) received only a few hundred thousand responses (retweets, likes and comments, etc.) at the height of the COVID-19 pandemic. Conversely, false information and information about conspiracy theories gained 52 million views (Mian and Khan, 2020). The amount of debunking information is always less than that passing on rumors. This means that the overall impact of debunking information is limited (Jung et al., 2020).

Further, researchers have found that most users who believe rumors do not take corrective action after reading the debunking information (Arif et al., 2017). Scholars who investigated the reactions of misinformed Twitter users after the users read debunking information found that they do not take any real action after seeing the debunking information (Wang and Zhuang, 2018). Consequently, rumors always spread faster and wider than debunking information, making it difficult for debunking information to curb the rumor spread and significantly reducing the effectiveness of debunking behavior (Vosoughi et al., 2018; Wang and Qian, 2021). It is important to investigate the behavior of sharing (referred to as *sharing behavior* hereon) debunking information; develop strategies that enable wider dissemination of debunking information; and form an effective countermeasure to curb the spread of rumors, mitigate the harm caused by them, and improve the efficiency of crisis management systems.

Five basic components of how individuals communicate with one another include *who*, *what*, through *which* channel, to *whom*, and with *what* effect (Lasswell, 1948). In the present study, from the perspective of the information publisher, the main concerns are the *who*, *what*, and *which* channel.

In the real world, people are concerned with the source of a story (McCracken, 1989). We accept statements from those we regard as experts (Hovland et al., 1953). Scholars have conducted empirical analyses of rumor development. The findings have suggested that rumors that are debunked by official sources are more likely to be arrested from continuing to spread (Andrews et al., 2016; Jung et al., 2020). Regarding the relationship between information sources and sharing behavior, Kim and Dennis (2019) found that highlighting the source of an article affects the extent to which readers trust the article, which in turn influences users’ engagement with the same (e.g., read, like, comment, and share). However, to the best of our knowledge, no further research has been conducted on the relationship between the identity of debunkers and information-sharing behavior. Previous research about the effect of type of source on sharing behavior has mostly used an experimental approach, studying the intention to share rather than the actual act of sharing. Using real data from social media, the relationship between debunker identity and sharing behavior in the context of social media can be studied with better ecological validity (Burton et al., 2021).

In this manuscript, the identity of the debunker refers to the type of Weibo account—that is, ordinary, celebrity, media, and government. This study focuses on the following question: How does the debunker’s identity affect the sharing behavior of information that is intended to debunk a rumor that has been spread through social media? It is clear that certain types of accounts have many followers. Accordingly, it is difficult to determine whether the behavior of reposting debunking information is influenced by the type of account or a higher number of followers.

Previous studies have confirmed the relationship between emotions and information dissemination. Messages with emotional overtones were forwarded more frequently and rapidly than neutral messages (Stieglitz and Dang-Xuan, 2013). Although negative messages spread faster than positive ones, positive messages reached larger audiences. This suggests that people prefer sharing positive content. This is known as positive bias (Ferrara and Yang, 2015). Moreover, some researchers have explored the influence of emotion on the spreading of rumors. For instance, Weeks (2015) and Martel et al. (2020) established that anger can promote belief in rumors and that emotional response increases belief in false news. This can make debunking difficult. Emotions play a significant role in the proliferation of both conventional information and rumors. However, debunking behaviors and debunking information have a specific set of characteristics that are less likely to gain attention and reposts than rumors (Arif et al., 2017; Wang and Zhuang, 2018). It is unclear whether the use of emotional language in debunking information increases the likelihood of reposting. Moreover, there are many potential problems with sentiment analysis of text data on social media. First, for data obtained from social media, pre-processing procedures such as removing deactivated words and removing account names or hashtags are usually needed at the initial stage, which do not have a strict standard; this may lead to different interpretations of the data (Burton et al., 2021). Second, the decision to use a lexical (or bag-of-words) approach or machine learning strategy in text classification may result in differences in the recognition of moral expressions in the same corpus, and classification performance may vary by context (Hoover et al., 2020). Finally, although computerized sentiment analysis allows researchers to test hypotheses on a larger dataset, it does not capture specific sentiments, such as sarcasm, which can render the final classification results biased (Stieglitz and Dang-Xuan, 2013). In view of the shortcomings of previous studies, this study first makes improvements to the sentiment analysis method to obtain more accurate sentiment classification results and then examines the relationship between text sentiment and sharing behavior in the context of social media.

Using the bootstrap method, this study examined the mediating role of follower count between three categories of social media accounts (celebrity, media, and government accounts) and the number of reposts, with the ordinary account as reference. A negative binomial regression model was used to examine the relationship between the sentiment expressed in debunking information and the number of reposts. We found that the relative mediating effect, or suppression effect of follower count, between these three types of accounts and sharing behavior was significant. The three account types had a significant positive effect on the number of reposts through the follower count. Further, after controlling for mediating variables, the debunker’s identity contributed little to the sharing of debunking information. In addition, debunking information with emotional overtones (positive or negative) was more likely to be reposted. However, the dominant emotional polarity varied in different rumor transmission contexts.

## Theoretical Bases and Hypotheses

### The Relationship Between the Debunker’s Identity and the Number of Reposts of the Debunking Information

The way we perceive the source shapes the way we think about subsequent information. We tend to view information from reputable sources positively and information from disreputable sources negatively; therefore, we are more likely to trust information from reputable sources (Tormala et al., 2006, 2007). Most debunking information posted by personal accounts is associated with news agencies and government organizations (Hunt et al., 2020). In addition, influential accounts on social media, such as media and celebrities, trigger more interactive behavior (Stieglitz and Dang-Xuan, 2013). Therefore, we propose the following hypothesis:

*H1a: Debunking information posted by celebrities, media, and government accounts will receive more reposts than information in ordinary accounts.*

### The Mediating Role of the Number of Followers of a Debunker

Scholars have found that compared to ordinary accounts, social media accounts that belong to celebrities, those that belong to media houses (e.g., Weibo-certified account of a newspaper, or a magazine), and those that belong to government organizations hold more reliable information (Andrews et al., 2016). Social media users, therefore, consult them because they are considered reliable and authoritative. The authority effect states that if a person is authoritative and respected, what they say and do is more likely to attract other people’s attention and to be believed. The prevalence of the authority effect is due to the psychological human desire to feel safe. People tend to believe that authorities mean well. Their statements are, accordingly, mostly believable (Scherman, 1993). Hence, following authorities on social media makes individuals feel safe. It also increases their faith in the authorities and the credibility of what they say. Moreover, it increases their confidence in their own credibility. Furthermore, most people tend to seek approval from prominent figures (Yuhong et al., 2019). They tend to believe that the words and actions of those in authority are in line with social norms. Accordingly, individuals who are consistent with authorities receive approval or praise from others. Therefore, we propose the following hypothesis:

*H1b: Celebrity, media, and government accounts will have more followers than ordinary account types.*

The number of followers a user has on social media represents the degree of homogeneity among their followers (Aral et al., 2009). This suggests that a user’s followers are likely to have similar interests. Therefore, they are more likely to repost the user’s content, leading to the following hypothesis:

*H1c: There is a positive correlation between the number of followers and the number of reposts.*

On the basis of H1a, H1b, and H1c, the influence of a debunker’s identity on information-sharing behavior can be divided into two premises: (1) the identity of the debunker directly affects the number of reposts, and (2) the identity of the debunker affects the number of reposts by influencing the number of followers. Thus, we propose the following hypothesis:

*H1: Using the ordinary account type as the reference, follower count is a relative mediating variable between celebrity, media, and government-type accounts and repost counts.*

### The Relationship Between Sentiment and the Number of Reposts of Debunking Information

The social contagion theory holds that individuals’ emotions and behaviors can be influenced by other people’s words, texts, expressions, gestures, and other messages (Kunitski et al., 2019). Users unconsciously spread positive and negative emotions through the comments they pass on to others through social networks. They trigger similar emotions and behaviors. People who use emotive language (including both positive and negative emotions) in their messages in social media forums receive more feedback than those who do not (Huffaker, 2010). Furthermore, research has also shown that users’ attraction to emotional content is not limited to a particular domain. This means that users tend to repost information that has a greater emotional impact, regardless of what the information is about (Milkman and Berger, 2014). We believe that this common rule also applies to the sharing behavior of debunking information. Hence, the following hypothesis is proposed:

*H2a: Debunking information with positive or negative sentiment is shared more often than the sharing of information with a neutral sentiment.*

Regarding the influence of positive and negative emotions on information-sharing behavior, some studies have shown that content that conveys positive emotions receives more attention and triggers higher levels of arousal, which can further influence feedback and social sharing behavior (Kissler et al., 2007; Berger, 2011; Bayer et al., 2012; Dang-Xuan and Stieglitz, 2012; Stieglitz and Dang-Xuan, 2013; Zollo et al., 2017). Therefore, we propose the following hypothesis:

*H2b: Debunking information with a positive sentiment is shared more often than that with a negative sentiment.*

## Materials and Methods

### Data Collection

We chose Sina Weibo because of its popularity in China and its unique “repost” feature as a powerful mechanism for sharing information (Pulido Rodríguez et al., 2020). In the context of Sina Weibo, users first post the original post-debunking information (information used to debunk misinformation). The original post is then disseminated to a new set of audience through re-posting, thus achieving the purpose of sharing and spreading the debunking information.

Through the Zhiwei Data Sharing Platform (Zhiwei Data, China) with Sina Info’s Enterprise Interface API, we collected data from posts on Sina Weibo between January 2020 and June 2021, carrying rumors that were eventually proven to be false. The three most widely spread rumors were as follows:

1. The Dragon Boat Festival, held by the University of Electronic Science and Technology (UESTC), was thought to invite Chinese female students to accompany international male students. This was later confirmed to be false information.
2. Mr. Yuan Longping was thought to have died while he was still receiving treatment in the hospital; this was later confirmed to be false information.
3. COVID-19 “Patient Zero” was thought to be a graduate student at the Wuhan Institute of Virology, which was later confirmed to be false information.

The dataset contained 4,586 original microblogs (489 posts about “UESTC,” 3 190 posts about “Yuan Longping,” and 907 posts about “Wuhan Institute of Virology”).

### Identification of Rumors and Debunking Messages

We grouped the relevant microblogs into five categories in line with Jung et al.’s (2020) classification. This included the following:

1. Rumor: false information is published, no doubt is expressed.
2. Debunking message: a rumor is denied, or a correction is published, the rumor is corrected in the post itself or through a linked article.
3. Uncertainty about rumor: the rumor is published, but it is questioned.
4. Uncertainty about debunking message: the debunking message is published, but it is questioned.
5. Others: jokes, unclear statements, and opinions.

We used manual tagging to filter the debunking information from the collected posts. First, three researchers in the field of social media (two Ph.D. students and one expert from Zhiwei Technologies Ltd.) annotated the 4,586 posts. We then used Cohen’s Kappa to ensure that the annotation scheme was consistent and valid. Next, we excluded *rumor*, *uncertainty about debunking message*, and *others* (1, 4, and 5 in the classification above), while retaining *debunking message* and *uncertainty about rumor* (2 and 3 above). Finally, 1,196 pieces of debunking information (including 304 of the UESTC, 447 of Yuan Longping, and 445 of the Wuhan Institute of Virology) were obtained.

### Identification of Account Roles and Debunking Message Types

Mirbabaie et al. (2014) identified five main backgrounds that debunkers may belong to. These included the following:

1. Emergency service organizations.
2. Media organizations (including journalists and bloggers).
3. Political groups and unions.
4. Individuals (political engagement or personal involvement).
5. Business organizations.

In this study, some adjustments were made to this classification method to consider microblog authentication types. The authenticated accounts were further divided into three categories:

1. Government
2. Media
3. Celebrity

Thus, debunkers were finally classified into the following four categories:

1. Ordinary accounts
2. Celebrity accounts
3. Media accounts
4. Government accounts

Three researchers examined the account types of debunkers for 1,196 debunked messages and ensured a consistent tagging scheme through Cohen’s Kappa.

### Sentiment Analysis

Sentiment analysis is a popular technique that is used to detect positive, neutral, or negative emotions from text. In this case, we sought to detect these emotions in social media content. For example, there are several algorithms specifically designed for short informal texts (Paltoglou and Thelwall, 2010; Hutto and Gilbert, 2014). Among these emotion analysis algorithms, Sentistrength is a promising one. The algorithm assigns positive *S*^+^(*t*) and negative *S*^−^(*t*) emotion scores to each piece of information and uses a single index to capture their polarity. That is, the emotional value *S*(*t*) is defined as the difference between positive and negative emotion scores.

However, there are some defects in the current emotion analysis methods for information on social media. The defects could decrease the accuracy of classification. For instance, the accuracy of Sentistrength in capturing positive emotions is only 60% (Ferrara and Yang, 2015). This may be caused by several factors, for example, the informality of the network text and the ambiguity of the same words used under different backgrounds. To overcome these shortcomings, this study calculated positive and negative emotionally charged words in each text based on the improved emotional dictionary. It took the difference between the two as the final emotional tendency. To calculate the emotional value, we used the following equation:


$$S\left(i\right)=S^{+}\left(i\right)-S^{-}\left(i\right),$$

where *S*(*i*) indicates the emotional value; *S*^+^(*i*) indicates the number of positive emotional words in the article *I*; and *S*^–^(*i*)indicates the number of negative emotional words in the article *i*.

We used the National Taiwan University Simplified Chinese Dictionary and the Simplified Chinese Emotion Dictionary of Taiwan University and made the following amendments to the dictionary based on the characteristics of each rumor.

1. Emotional symbols play an important role in emotional expression. We thus converted the emotional symbols in the text into machine-recognizable words and incorporated them into the emotional value calculation.
2. For different rumors, we randomly selected one-third of the texts, analyzed their expression characteristics, and added words with obvious satire and ridicule to the dictionary.
3. When debunking a rumor, it is necessary to first describe it. In this context, the emotional words in the rumor cannot represent the emotional tendency of the debunking information. Therefore, we made some adjustments. The emotional words in the original rumor were not calculated as the emotional value of the debunking information. Based on the above improvements, we used Python to calculate the emotional polarity of each text and labeled it as positive, middle, or negative according to its emotional polarity value.

## Results

### Preliminary Analysis

All statistical analyses were performed using Stata version 16.1. Samples 1, 2, and 3 represent rumors 1, 2, and 3, respectively. The sample number, mean, standard deviation, and maximum and minimum values of the major variables in the three rumor samples are shown in Table 1.

**TABLE 1:** Descriptive statistics of the major variables.

Sample	Variable	Obs	Mean	Std. Dev.	Min	Max
Sample 1	Y	304	9.586	79.99	0	1,240
	ac1	304	0.273	0.446	0	1
	ac2	304	0.151	0.359	0	1
	ac3	304	0.046	0.21	0	1
	s1	304	0.349	0.477	0	1
	s2	304	0.023	0.15	0	1
	Fol	304	1212264.9	6892123.9	2	1.03E+08
Sample 2	Y	447	40.497	283.375	0	4,625
	ac1	447	0.28	0.449	0	1
	ac2	447	0.125	0.331	0	1
	ac3	447	0.02	0.141	0	1
	s1	447	0.805	0.396	0	1
	s2	447	0.051	0.221	0	1
	Fol	447	2091211.9	9602701.3	0	1.03E+08
Sample 3	Y	445	6.865	66.674	0	1,088
	ac1	445	0.227	0.419	0	1
	ac2	445	0.658	0.475	0	1
	ac3	445	0.038	0.192	0	1
	s1	445	0.083	0.276	0	1
	s2	445	0.382	0.486	0	1
	fol	445	5442990.3	13975008	1	1.20E+08

*Y represents the number of reposts.*

*ac1–ac3 represent celebrity-, media-, and government-type accounts, respectively, 0 or 1.*

*s1 and s2 represent negative and positive sentiment, respectively, 0 or 1.*

*Fol represents the number of followers.*

The preliminary basic statistics of sample 1 revealed 304 observations, and the average number of reposts was 9,586. The proportions of celebrities, media, and government accounts were 27.3, 15.1, and 4.6%, respectively. Positive emotion accounted for 34.9%, and negative emotion accounted for 2.3% of the sample. In sample 2, there were 447 observations, and the average number of reposts was 40,497. The proportions of celebrities, media, and government accounts were 28.0, 12.5, and 2.0%, respectively, and the proportions of positive and negative emotions were 80.5 and 5.1%, respectively. In sample 3, there were 445 observations, and the average count of reposts was 6,865. The proportions of celebrities, media, and government accounts were 22.7, 65.8, and 3.8%, respectively, and the proportions of positive and negative emotions were 8.3 and 38.2%, respectively.

### Mediation Model Testing

We used the bootstrap approach to test for mediating effects. The bootstrap test is one of the coefficient product tests among the mediating effect tests. It was the most common mediating effect test at the time of this study. It is based on the theoretical concept of standard error, which treats the large-size sample as the total and conducts put-back sampling to obtain a more accurate standard error (Biesanz et al., 2010). The independent variable is the type of account (divided into ordinary, celebrity, media, and government), which is a four-category independent variable. The mediating variable was the number of followers, and the dependent variable was the number of reposts. Since dependent variables take a wide range of values, they can be treated as continuous variables.

Using the ordinary account-type as reference, we examined the mediating role of follower count between celebrity, media, and government accounts and the number of reposts (Fang et al., 2017).

**FIGURE 1:**
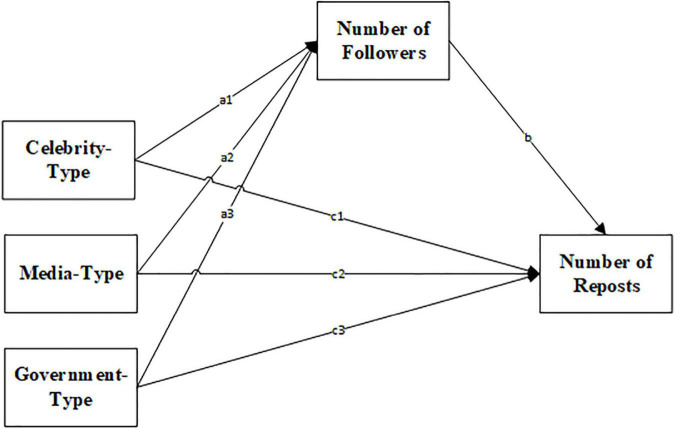
The mediation mode. a × b represents the value of the indirect effect (which is the value of ind_eff in Table 2), c represents the value of the direct effect (which is the value of dir_eff in Table 2). For example, in Sample 1, the indirect effect a1 × b of the celebrity-type account on the number of reposts is ind_eff = 0.457 and is significant at the 1% level, and the direct effect c1 is dir_eff = 0.054 and is not significant.

A number of factors influence the sharing behavior, such as text sentiment, message tagging, whether it contains a URL, whether it contains an image, and whether it contains a video (Stieglitz and Dang-Xuan, 2013; Ruths and Pfeffer, 2014; Ferrara and Yang, 2015; Brady et al., 2017; Howard et al., 2018; Lazer et al., 2018). Unlike regular information, debunking information has its own characteristics. Some studies have shown that the more quickly a rumor is debunked, the more effective the debunking was (Jung et al., 2020). Therefore, we added the delay in posting the debunking information as a control variable. Additionally, we included the following variables as control variables: text sentiment, whether the message contained a tag, whether the message contained a URL, whether the message contained an image, and whether the message contained a video. The mediation effect model is shown in Figure 1.

Table 2 shows the results of the mediation effects test using the bootstrap approach for the three rumor samples.

**TABLE 2:** Results of bootstrap.

	Var	Effect	Coef.	S.E.	*z*	95% CI (BC)
Sample1	ac1	ind_eff	0.457	0.133	3.43***	[0.244, 0.784]
		dir_eff	0.054	0.149	0.36	[–0.224, 0.342]
	ac2	ind_eff	0.634	0.177	3.58***	[0.338, 1.070]
		dir_eff	0.181	0.26	0.69	[–0.305, 0.715]
	ac3	ind_eff	0.289	0.105	2.75***	[0.142, 0.584]
		dir_eff	0.392	0.312	1.26	[–0.150, 1.079]
Sample2	ac1	ind_eff	0.604	0.13	4.66***	[0.387, 0.908]
		dir_eff	–0.196	0.105	–1.87	[–0.410, 0.004]
	ac2	ind_eff	1.137	0.25	4.54***	[0.687, 1.701]
		dir_eff	0.695	0.397	1.75	[–0.055, 1.525]
	ac3	ind_eff	0.845	0.242	3.49***	[0.438, 1.448]
		dir_eff	0.009	0.673	0.01	[–1.149, 1.552]
Sample3	ac1	ind_eff	0.797	0.177	4.51***	[0.515, 1.213]
		dir_eff	–0.52	0.205	–2.54**	[–0.972, –0.173]
	ac2	ind_eff	0.996	0.215	4.63***	[0.631, 1.487]
		dir_eff	–0.711	0.201	–3.54***	[–1.146, –0.343]
	ac3	ind_eff	0.634	0.166	3.83***	[0.360, 1.012]
		dir_eff	–0.671	0.244	–2.75***	[–1.132, –0.173]

***p < 0.01; **p < 0.05; *p < 0.1.

*ac1-ac3 represent celebrity-, media-, and government-type accounts, respectively, 0 or 1.*

*ind_eff represents the value of the indirect effect.*

*dir_eff represents the value of the direct effect.*

In sample 1, the relative direct effects of the three account types—celebrity, media, and government—on the number of reposts were not significant. The relative indirect effects on the number of reposts were significant (a1 × b = 0.457, *p* < 0.01; a2 × b = 0.634, *p* < 0.01; a3 × b = 0.289, *p* < 0.01). In sample 2, the relative direct effects of the three account types on the number of reposts were not significant. The relative indirect effects on the number of reposts were significant (a1 × b = 0.604, *p* < 0.01; a2 × b = 1.137, *p* < 0.01; a3 × b = 0.845, *p* < 0.01). Thus, in samples 1 and 2, the mediating effect of follower count on the relation between the three types of accounts—celebrity, media, and government—and number of reposts was significant, using the ordinary account type as reference. In sample 3, the relative direct effect of the three account types on the number of reposts (c1′ = –0.52, *p* < 0.01; c2′ = –0.711, *p* < 0.01; c3′ = –0.671, *p* < 0.01) was significant, and the relative indirect effect on the number of repost (a1 × b = 0.797, *p* < 0.01; a2 × b = 0.996, *p* < 0.01; a3 × b = 0.634, *p* < 0.01) was also significant, and the indirect and direct effects are shown as opposite signs. In sample 3, the suppression effect of follower count between the three types of accounts and the number of reposts was significant, using the ordinary account type as reference. Suppression means the total effect was masked when indirect and direct effects were significant and opposite (MacKinnon et al., 2002; MacKinnon, 2008).

Therefore, we can conclude that the number of followers plays a mediating, or a suppressive, role between the three account types (celebrity, media, and government) and the number of reposts when the ordinary account type is used as the reference level. Thus, H1 was verified.

### Regression Analysis

#### Negative Binomial Regression Analysis

To test H2a and H2b, which state that debunking information with emotional overtones (positive or negative) receive more reposts than neutral messages and that debunking information with positive sentiment is shared more often than those with negative sentiment, respectively, the following variables were constructed: as the dependent variable, the amount of reposted debunking information; as the independent variable, the emotional polarity of the debunking information.

We included the following as control variables: whether the account was certified, whether the message contained a tag, whether the message contained a URL, whether the message contained an image, whether the message contained a video, and the time difference between rumor and debunking information.

We used a regression model to test H2a and H2b. As our dependent variable represented the number of reposts of one piece of information, which was a non-negative integer, and the variance of the number of times it was forwarded in the three samples was far greater than the mean value, as shown in Table 1, we used a negative binomial regression model for all the three samples. The results are shown in Tables 3–5.

**TABLE 3:** Negative binomial regression results: sample 1.

y	Coef.	S.E.	*t*-value	95% CI
s1	751	0.434	1.73*	[–0.101, 1.602]
s2	2.289	0.592	3.86***	[1.128, 3.45]
d_std	–0.766	0.184	–4.16***	[–1.127, -0.405]
lnfol	0.214	0.06	3.59***	[0.097, 0.331]
ac	0.758	0.503	1.51	[–0.227, 1.743]
url	1.349	0.749	1.80*	[–0.118, 2.817]
tag	2.045	0.617	3.32***	[0.836, 3.254]
pic	1.659	0.458	3.63***	[0.762, 2.556]
vid	–0.49	0.671	–0.73	[–1.804, 0.824]
Constant	–5.002	0.742	–6.74***	[–6.457, –3.548]
lnalpha	1.898	0.179		[1.548, 2.249]
Mean dependent var		9.586	SD dependent var	79.990
Pseudo *r*-squared		0.138	Number of obs	304
Chi-square		148.790		0.000

***p < 0.01; **p < 0.05; *p < 0.1.

*s1 and s2 represent negative and positive sentiment, respectively, 0 or 1.*

*d_std, lnfol, ac, url, tag, pic, and vid are all control variables, where d_std represents the standardized delay in posting the debunking information.*

*lnfol represents the number of followers after taking logarithms.*

*ac represents whether the account is authenticated, 0 or 1.*

*url represents whether the message include URL, 0 or 1.*

*tag represents whether the message include tag, 0 or 1.*

*pic represents whether the message include pictures, 0 or 1.*

*vid represents whether the message include video,0 or 1.*

**TABLE 4:** Negative binomial regression results: sample 2.

y	Coef.	S.E.	*t*-value	95% CI
s1	1.738	0.455	3.82***	[0.847, 2.63]
s2	2.377	0.74	3.21***	[0.926, 3.828]
d_std	–0.902	0.212	–4.26***	[–1.317, –0.487]
lnfol	0.747	0.07	10.65***	[0.61, 0.885]
ac	–0.051	0.586	–0.09	[–1.199, 1.097]
url	–0.738	0.471	–1.57	[–1.662, 0.186]
tag	–0.333	0.63	–0.53	[–1.567, 0.902]
pic	–0.197	0.463	–0.42	[–1.105, 0.711]
vid	–1.022	0.623	–1.64	[–2.244, 0.2]
Constant	–8.361	0.932	–8.97***	[–10.188, –6.533]
lnalpha	1.852	0.136		[1.585, 2.119]
Mean dependent var		40.862	SD dependent var	284.628
Pseudo *r*-squared		0.188	Number of obs	447
Chi-square		484.377		0.000

***p < 0.01; **p < 0.05; *p < 0.1.

*s1 and s2 represent negative and positive sentiment, respectively, 0 or 1.*

*d_std, lnfol, ac, url, tag, pic, and vid are all control variables, where d_std represents the standardized delay in posting the debunking information.*

*lnfol represents the number of followers after taking logarithms.*

*ac represents whether the account is authenticated, 0 or 1.*

*url represents whether the message include URL, 0 or 1.*

*tag represents whether the message include tag, 0 or 1.*

*pic represents whether the message include pictures, 0 or 1.*

*vid represents whether the message include video, 0 or 1.*

**TABLE 5:** Negative binomial regression results: sample 3.

y	Coef.	S.E.	*t*-value	95% CI
s1	2.526	0.786	3.21***	[0.986, 4.065]
s2	0.942	0.438	2.15**	[0.083, 1.801]
d_std	–.664	0.224	–2.97***	[–1.103, –0.226]
lnfol	0.67	0.091	7.37***	[0.492, 0.848]
ac	–2.638	1.12	–2.36**	[–4.833, –0.443]
url	–0.098	0.403	–0.24	[–0.889, 0.693]
tag	1.032	0.381	2.71***	[0.285, 1.78]
pic	–0.388	0.366	–1.06	[–1.106, 0.33]
vid	0.117	0.112	1.04	[–0.103, 0.337]
Constant	–7.319	1.011	–7.24***	[–9.3, –5.337]
lnalpha	2.162	0.136		[1.894, 2.429]
Mean dependent var		6.865	SD dependent var	66.674
Pseudo *r*-squared		0.101	Number of obs	445
Chi-square		98.614		0.000

***p < 0.01; **p < 0.05; *p < 0.1.

*s1 and s2 represent negative and positive sentiment, respectively, 0 or 1.*

*d_std, lnfol, ac, url, tag, pic, and vid are all control variables, where d_std represents the standardized delay in posting the debunking information.*

*lnfol represents the number of followers after taking logarithms.*

*ac represents whether the account is authenticated, 0 or 1.*

*url represents whether the message include URL, 0 or 1.*

*tag represents whether the message include tag, 0 or 1.*

*pic represents whether the message include pictures, 0 or 1.*

*vid represents whether the message include video, 0 or 1.*

The regression results showed that in sample 1, both negative and positive sentiments had a positive effect on the reposting of information compared to a neutral sentiment (positive: coef = 2.289, SE = 0.592, *p* < 0.01; negative: coef = 0.751, SE = 0.434, *p* < 0.1). Moreover, we observed that the coefficient of positive sentiment was 3.05 times higher than that of negative sentiment. These results demonstrate that positive or negative debunking information had a higher number of reposts, compared to neutral sentiment, and that positive sentiment had a boosting effect on the number of reposts in sample 1. In sample 2, we repeated the process above. The results from sample 2 were similar to those from sample 1. Specifically, both positive and negative sentiment received more reposts, relative to neutral sentiment (positive: coef = 2.377, SE = 0.74, *p* < 0.01; negative: coef = 1.738, SE = 0.455, *p* < 0.01). The coefficient value of positive sentiment was observed to be 1.37 times higher than negative sentiment. In sample 3, the facilitation effect of positive or negative sentiment on information forwarding, relative to neutral sentiment, remained significant (positive: coef = 0.942, SE = 0.592, *p* < 0.05; negative: coef = 2.526, SE = 0.786, *p* < 0.01). However, unlike samples 1 and 2, we found that the coefficient of positive sentiment was 0.373 times higher than the coefficient of negative sentiment; that is, negative sentiment had a greater impact than positive sentiment on the sharing behavior of debunking information. Therefore, H2a was accepted, while H2b was not accepted.

#### Model Robustness Tests

We further corroborated our findings by excluding alternative explanations and checking for robustness and consistency in a number of ways.

First, there may be concerns about the potential presence of heteroscedasticity, which could bias the standard errors of the estimates. Therefore, we used heteroscedasticity robust standard errors in all models.

Second, we checked the robustness of our findings using different models. We re-estimated using OLS and obtained results that were consistent with those in Table 2.

Finally, we investigated whether our findings were robust under different combinations of control variables. Therefore, we added gender and text length as new control variables and found that the results obtained in Table 2 were consistent.

In summary, all tests indicate that our findings are robust and consistent.

#### Model Endogeneity Issues

The endogeneity of the model is not a serious problem owing to several reasons:

1. The independent and dependent variables have a clear chronological order, with the message being shared first and the reposts after, with no reverse causality.
2. The independent variable, text sentiment, is determined by the information publisher, while the dependent variable is determined by the forwarder, making it more difficult to have a third variable that affects both simultaneously, leading to pseudo-causality.
3. The use of data from the same sample in the same regression model prevents the appearance of factors that may affect both independent and dependent variables owing to the character of the rumor topic.

## Discussion

### Discussion of the Results

First, using the ordinary account type as the reference, we observed a significant mediating or suppression effect of followers between the three types of accounts (celebrity, media, and government) and sharing behavior. In all the samples, these three account types had a significant positive effect on the number of reposts through the number of followers. The debunker’s identity did not promote the sharing of debunking information while controlling for mediating variables. Information was shared to meet certain needs, and these needs motivated sharing the information in line with Tellis et al.’s (2019) findings. According to the hierarchical theory of needs, the lower the level of a need, the greater the effort of an individual paid for satisfying the need (Maslow, 1943, 1970, 1987). Specifically, in samples 1 and 2, the relative indirect effect of follower count between the three account types and sharing behavior was significantly positive, while the relative direct effect of account type on the number of reposts was not significant. Regarding rumor propagation in samples 1 and 2, people’s needs, such as socialization and entertainment, fell in the belongingness level of Maslow’s hierarchy of needs. In this context, users did not invest much energy in debunking information. The direct effect of the debunker’s identity on sharing behavior was not significant. We believe that this was largely due to the oversight of sources—the information presented on social media was different from traditional media platforms.

In traditional media (e.g., TV news, newspapers, and news websites), the audience knows about the source of the information before they view the content. This affects how the audience treats the subsequent information. However, on social media, users do not choose a source of news. They get cocktails of stories from several different sources containing posts shared by friends, articles from sources the users have read before, and articles from sources users have not chosen. These posts could be real or fake, with the intention of deliberately influencing users’ opinions and actions (Kim and Dennis, 2019). Moreover, some studies suggest that the current design of social media platforms—where users present their immediate feedback after quickly scrolling through formal news or emotional content—may block people’s minds from thinking about additional factors, such as the reliability of the source. This influences users’ sharing behavior (Pennycook et al., 2021). Thus, when the information meets the needs of the user, the confusing source of debunking information combined with the unique way in which users navigate information on social media will cause the user to focus primarily on the information. This could mean that the source has less influence on the sharing behavior.

In sample 3, the relative indirect effect of follower count between the three account types and sharing behavior was significantly positive, and the relative direct effect of account type on the number of reposts was significantly negative. We believe that this was due to the unique context of the rumor in sample 3. Unlike the circumstances of rumors in samples 1 and 2, in the context of a sudden public crisis, such as the COVID-19 outbreak, people’s needs are concentrated at the physiological and safety needs levels. These are more powerful than belongingness needs and above. Hence, people would invest more energy in debunking information, leading to increased attention to sources of information. However, some studies suggest that when denied rumors were later proved to be factual information, users would reduce their trust in similar denials in the future (Wang and Huang, 2021). Users’ beliefs about the information itself (i.e., confirmation bias) affect their perception of the source (Tormala et al., 2006, 2007). During the spread of the rumor regarding the Wuhan Institute of Virology, the early false denial of COVID-19 created distrust in the so-called “authoritative channels.” Here, the special identity of the debunker (celebrity, media, and government) becomes a hindrance to the sharing of debunking information. Thus, to increase the forwarding of debunking information on social media, the first step is to expand the potential audience—that is, the number of followers—rather than emphasizing the debunker’s identity, which is commonly treated as a crucial factor.

Second, we found that debunking information with positive or negative emotions was forwarded more frequently than that with neutral emotions. This was consistent with previous studies that demonstrated the role of emotion in information diffusion (Bell et al., 2001; Huffaker, 2010; Berger and Milkman, 2012; Milkman and Berger, 2014). Our research confirmed that this finding also applies to the sharing of debunking information. Our findings were different from the findings of Ferrara and Yang (2015) and Zollo et al. (2017), who reported that information with positive sentiment always received more reposts. We found that it was not certain that information with positive emotions would always be shared more than that with negative emotions. We found that debunking information that was laced with negative emotions could be shared more: for example, in samples 1 and 2, positive emotions played a greater role, while in sample 3, negative emotions played a greater role. When users read the information, the emotion in it is perceived by them in two ways. One is perception of the emotion expressed by the debunking information, and the other is formed by the people’s first impression of the subject of the rumor. The subjects of the three rumors in this study were the UESTC, Mr. Yuan Longping, and the Wuhan Institute of Virology. UESTC is a prestigious university in China, and Mr. Yuan Longping is a world-renowned expert in hybrid rice, but Wuhan Institute of Virology has a negative public reputation owing to some previous mishaps, such as the widespread mistrust caused by its fake announcement that “Shuanghuanglian” could treat COVID-19. The bias effect indicates that when people subjectively support a certain point of view, they tend to search for information that supports it as well and ignore the opposing view. The bias effect will cause an anchoring effect; that is, people prioritize their first impression while making decisions or judgments. Therefore, we believe that the first impression of the subjects in rumors may affect the forwarding behavior of information containing different emotional tendencies. When people perceive that the image of the subject tends to be positive, debunking information containing positive emotions such as blessings, encouragement, and praise conforms to people’s first impression of the subject and is easily recognized and forwarded. When the subject’s image tends to be negative, it is difficult to obtain people’s trust for the debunking information that expresses positive emotions due to the bias effect. People may tend to repost information that contains negative sentiments such as warnings, condemnation, and mockery of the person who posted the rumor. It would be interesting to empirically test this hypothesis in future studies. The positive correlation between emotion and forwarding frequency of debunking information has highlighted the significance of using emotional expression for debunking information to obtain more reposts. Further consideration of the use of positive or negative sentiment in a particular situation may be required.

### Implications

From a theoretical perspective, this study provides a conceptually grounded and empirically tested mediation model and a negative binomial regression model to explain the influence of a debunker’s identity and the emotional content in the message on the sharing behavior of debunking information on social media (Sina Weibo). It attempts to bridge a gap in research on the behavior of debunking information on social media.

From a practical perspective, the study has important implications for Chinese social media. It is especially useful to agencies involved with emergency response agencies for its insights on the packaging and management of debunking information on social media, for wider reach and expanded impact. From the perspective of “who”—the debunkers’ identity—previous research found that the source of an article affected the extent to which readers trusted it. This in turn influences users’ engagement with the article (they may, for example, decide to read, like, comment, and share). However, the finding was based on laboratory experiments, which might have generalizability issues in the real world (Kim and Dennis, 2019). In this study, we used actual data to test the hypotheses. We followed actual acts of sharing of real data on social media. After controlling the mediating variables (number of followers), we found that the identity of the debunker did not improve the sharing behavior. This may be due to the unique way in which information is presented on social media. The implications for those involved with emergency services are that when debunking information on social media, expanding the audience and engaging the public are the most reliable focal points.

Second, from the perspective of “what”—the content of debunking information—the emotional value of the message will influence the extent to which it is shared (Brady et al., 2017). We found that information that was laced with emotional value always received more reposts. This finding has implications for the content generation of debunking information. Those involved with debunking rumors on social media will benefit from this finding. While the finding on emotional messaging is not novel, we have confirmed its value in debunking rumors on social media.

### Limitations and Future Directions

This study has some limitations. First, our analyses were based on data from only three rumor spreads, which may raise the issue of overgeneralization, However, given that Weibo is a very popular social media platform and the rumors we selected were widespread, the issue the findings pass the test of generalization. Future studies could, however, validate the findings further. Second, only one of the three rumors involved a sudden public crisis event. Accordingly, the impact of the debunker’s identity on the sharing behavior in a sudden public crisis event requires further in-depth research. For example, using the ordinary account as a reference, it remains to be seen in what scenarios the number of followers has a mediating effect and in what scenario it has a suppression effect. Finally, this study did not investigate the reasons for the different effects of the same affective polarity (positive or negative) on sharing behavior in the context of different rumors. Future research could focus on the underlying reasons for the variability in the mechanisms by which emotions influence sharing behavior in different contexts.

## Conclusion

This manuscript advances research on debunkers’ identity and information sharing behavior through the number of followers as a mediating variable. Related to this is how emotions in debunking information affect reception of the message and message sharing. It makes useful contribution for strategic consideration by those involved with debunking rumors on social media.

## Data Availability Statement

The raw data supporting the conclusion of this article will be made available by the authors, without undue reservation.

## Ethics Statement

Ethical review and approval was not required for the study on human participants in accordance with the local legislation and institutional requirements. Written informed consent for participation was not required for this study in accordance with the national legislation and the institutional requirements.

## Author Contributions

FC: study conception, data analysis, and write the manuscript. XW: contribution to study conception. GY: study conception, contribution to data analysis and the manuscript. All authors have approved the final version of the manuscript and its submission.

## Conflict of Interest

The authors declare that the research was conducted in the absence of any commercial or financial relationships that could be construed as a potential conflict of interest.

## Publisher’s Note

All claims expressed in this article are solely those of the authors and do not necessarily represent those of their affiliated organizations, or those of the publisher, the editors and the reviewers. Any product that may be evaluated in this article, or claim that may be made by its manufacturer, is not guaranteed or endorsed by the publisher.
